# Improvement of Physico-mechanical Properties of Partially Amorphous Acetaminophen Developed from Hydroalcoholic Solution Using Spray Drying Technique

**Published:** 2013-10

**Authors:** Fatemeh Sadeghi, Mansour Torab, Mostafa Khattab, Alireza Homayouni, Hadi Afrasiabi Garekani

**Affiliations:** 1 Targeted Drug Delivery Research Center, School of Pharmacy, Mashhad University of Medical Sciences, Mashhad, Iran; 2 Department of Pharmaceutics, School of Pharmacy, Mashhad University of Medical Sciences, Mashhad, Iran; 3 Pharmaceutical Research Center, School of Pharmacy, Mashhad University of Medical Sciences, Mashhad, Iran

**Keywords:** Acetaminophen, Amorphous particles, Compaction, Crystallinity, Dissolution, Spray drying

## Abstract

***Objective(s): ***This study was performed aiming to investigate the effect of particle engineering via spray drying of hydroalcoholic solution on solid states and physico-mechanical properties of acetaminophen.

***Materials and Methods:*** Spray drying of hydroalcoholic solution (25% v/v ethanol/water) of acetaminophen (5% w/v) in the presence of small amounts of polyninylpyrrolidone K30 (PVP) (0, 1.25, 2.5 and 5% w/w based on acetaminophen weight) was carried out. The properties of spray dried particles namely morphology, surface characteristics, particle size, crystallinity, dissolution rate and compactibility were evaluated.

***Results:*** Spray drying process significantly changed the morphology of acetaminophen crystals from acicular (rod shape) to spherical microparticle. Differential scanning calorimetery (DSC) and x-ray powder diffraction (XRPD) studies ruled out any polymorphism in spray dried samples, however, a major reduction in crystallinity up to 65%, especially for those containing 5% w/w PVP was observed. Spray dried acetaminophen particles especially those obtained in the presence of PVP exhibited an obvious improvement of the dissolution and compaction properties. Tablets produced from spray dried samples exhibited excellent crushing strengths and no tendency to cap.

***Conclusions:*** The findings of this study revealed that spray drying of acetaminophen from hydroalcoholic solution in the presence of small amount of PVP produced partially amorphous particles with improved dissolution and excellent compaction properties.

## Introduction

The importance of the solid-state structure with regards to the physico-mechanical properties of pharmaceuticals has already been reported (1-3). Preparation of amorphous form is a possible approach to improve the physico-mechanical behaviors of pharmaceutical solids. Compared to a crystalline solid, the amorphous material can have advantages such as enhanced dissolution rate, bioavailability and tableting properties (4, 5). Amorphous solids can be produced by common pharmaceutical processes including melt quenching, super critical fluid, freeze drying, long period milling and spray drying (6). Spray drying of solutions is known as the most important industrial method to facilitate the production of amorphous material. 

Due to poor solubility of acetaminophen in water, spray drying of its aqueous suspensions has been widely used in pharmaceutical industry to improve its physico-mechanical properties (7, 8). Spray drying of a material from suspension often yields crystalline products due to crystalline material that remains in suspension. In fact, spray dried particles obtained from a suspension are agglomerates of original crystals, while spray drying the solution is a crystallization process which could lead to production of partially or completely amorphous materials due to rapid solidification of droplets (9). 

Many amorphous solids are thermodynamically unstable and can spontaneously transform into a crystalline state if exposed to sufficient moisture and heat (10, 11). It has been shown that addition of some polymers, known as stabilizing polymer, such as PVP, can prevent or minimize such these transformations (12-14). This effect has been attributed to adsorption of PVP onto the surfaces of the drug crystals. 

The production of amorphous acetaminophen by spray drying technique from aqueous solution in the presence of 50% w/w chitosan (based on acetaminophen weight) has already been reported (15). However, the use of high amount of polymer (more than 50%) and low drug concentration in dispersions have been the drawback for this study.

Garekani *et al* showed that PVP is an effective additive during crystallization of acetaminophen and significantly changed the crystal habit and physico-mechanical properties of obtained particles by adsorption onto the surfaces of acetaminophen crystals via hydrogen bonding (16-19). Therefore, PVP may act as an effective additive to improve both stability and physico-mechanical properties of acetaminophen during spray drying. 

In the present study, spray drying of acetaminophen is carried out from hydroalcoholic solution in the presence of small amounts of PVP, maximum 5% w/w based on acetaminophen weight. The solid states and physico-mechanical properties of spray dried particles were also investigated. To the best of our knowledge, there is no study regarding the use of hydroalcoholic solution for spray drying of acetaminophen. 

The use of hydroalcoholic solution for spray drying of acetaminophen has several advantages. Ethanol is one of the safest and cheapest organic solvents for acetaminophen. Solubility of acetaminophen in ethanol is 10 times higher than water (20). The solubility of acetaminophen in hydroalcoholic solution (25% V/V) is 50 mg/ml and therefore a solution with rather high solid content can be obtained. Besides, during spray drying process, this solvent is much less flammable compared to pure ethanol. Also the evaporation rate of hydroalcoholic solution is higher than water. Therefore, due to faster solidification during spray drying, the production of amorphous material is facilitated. 

## Materials and Methods

***Materials***

Acetaminophen was obtained from Temad Co., Iran. PVP-K30 was obtained from BASF, Germany. Ethanol 96% was obtained from Zakaria Co, Iran.

***Methods***

Hydroalcoholic solutions (25% v/v ethanol/water) of acetaminophen (5% w/v) were prepared in the presence of different amounts of PVP K30 (0, 1.25, 2.5 and 5%w/w, based on acetaminophen weight). These solutions were spray dried under the obtained optimal conditions of inlet drying air of 160 °C, outlet drying air of 85 °C, feed rate of 45 g/min and atomizing air pressure of 0.5 bar. Spray drying of these solutions was performed using a Mini Spray Dryer B-290, (BUCHI, Switzerland). The solutions were fed through a two fluid pressure atomizers at the top of the spray dryer by means of a peristaltic pump. The spray dryer was operated in co-current air flow and the powder was collected using a cyclone. 

***Process yield of spray drying***

After spray drying process, the production yield was calculated using the following equation:

$\mathrm{\% process Yield}=\left(\frac{\mathrm{Weight of obtained sample}}{\mathrm{Total solid content of each formulation}}\right)\times 100$           (Equation 1)

***Particle size measurement***

Optical microscope (Olympus BX60, Japan) was used in order to determine the size of particles. Tiny amounts of spray dried samples were spread on glass slides and Martin’s diameter of minimum 100 particles was measured using a micrometer fitted on eyepiece. 

***Scanning electron microscopy (SEM)***

Electron micrographs of acetaminophen particles were obtained using a scanning electron microscope (Oxford S360, UK). Voltage of 15 kV was selected for accelerating the electrons from electron gun onto the specimen. The specimens were mounted on a metal stub with double side adhesive tape and coated with gold in an argon atmosphere using Sputter Coater SC 7620, prior to observation. 

***Assessment of crystallinity of spray dried samples***

*Differential scanning calorimetry (DSC)*

A differential scanning calorimeter (Mettler Toledo DSC 822, Switzerland) was used to determine the melting points and fusion enthalpy of samples. The equipment was calibrated using indium. Acetaminophen samples (3-5 mg) were heated at 10°C/min in sealed aluminum pans under nitrogen atmosphere. The melting points and enthalpies of fusion of samples were calculated by the instrument. Percentage of relative crystallinity of samples ​​was calculated by the following equation (15).

 % Relative crystallinity = (ΔH_S _/ ΔH_R_)100                    (Equation 2)

**Table 1:** The spray drying process yield and mean particle size of samples obtained from solutions containing different amounts of polyninylpyrrolidone

Spray dried acetaminophen in the presence of PVP (%w/w)	Process yield (%)	Mean particle size (µm)
0	35.7 ± 5.1	11.0 2.1
1.25	43.4 ± 4.3	11.6 3.1
2.5	51.5 ± 6.5	14.2 3.3
5	65.1 ± 3.3	18.5 3.7
		

Here, H_S _is the enthalpy of fusion of spray dried sample and ΔH_R_ is the enthalpy of fusion of pure crystalline untreated acetaminophen.

*X-ray powder diffraction (XRPD)*

X-ray diffraction spectra of acetaminophen samples were obtained using a Philips X-ray diffractometer PW 1480, USA. The scanning rate of 1° 2θ/min over the range of 1–50° 2θ was used to obtain each spectrum. Percentage of relative crystallinity of samples was calculated by the following equation (21).

% Relative crystallinity = (I_S _/ I_R_) Χ 100           (Equation 3)

Here, I_S_ is the area under a distinct peak exactly at 18° 2θ in spray dried samples and I_R_ is the area under a peak at the same position in pure crystalline untreated acetaminophen. The peak at 18° 2θ is the largest and most distinguished peak in X-ray diffraction spectra of acetaminophen.

***Dissolution test***

Dissolution tests were carried out using an automated dissolution tester (Pharmatest, Germany). In all tests an appropriate amount of powder, equivalent to 40 mg of acetaminophen, was weighed and dusted on the dissolution medium (900 mL water) using USP apparatus II (Paddle method), rotating at 50 rpm. Filtered samples were taken from the vessels by a peristaltic pump (Alitea, Sweden) at different intervals, and assayed at 242 nm by a multi-cell transport spectrophotometer (Shimadzu, Japan) based on calibration curve obtained for acetaminophen at this wavelength. 

Mean dissolution time (MDT) was calculated for each formulation using the following equations and mean and standard deviation were determined. 

$\mathrm{MDT}=\sum t_{i}.∆M_{i}/\sum ∆M_{i}$           (Equation 4)

$t=(t_{i}+t_{i+1})$/2           (Equation 5)

$∆M_{i}=(M_{i+1}-M_{i})$          (Equation 6)

Where t*¯* is the midpoint of the time period during which the fraction *M *of the drug was released from sample.

***Compaction study***

Acetaminophen samples were compacted using an instrumented single punch tableting machine (Korsch, Germany) fitted with 8 mm flat faced punches. The die wall was prelubricated with 4% w/w magnesium stearate in acetone before each compression. Accurately weighed amount of 120 mg samples were hand filled into the die. At least 10 tablets were produced at each compression forces of 5, 10 and 15 kN. For making comparison, the physical mixtures of acetaminophen and sieved fraction (<45 µm) of PVP (2.5 or 5% w/w, with respect to acetaminophen weight) were also prepared and compressed at the above-mentioned different compression forces. Tablet hardness was measured using a tablet hardness Tester (Erweka TBH200, Germany). Mean hardness of 5 tablets from each formulation was reported as tablet hardness. 

## Results

***Process yield and particle size***

The percentage yield of spray drying process is presented in Table 1. 

***Appearance of*** ***particles***

**Figure 1:**
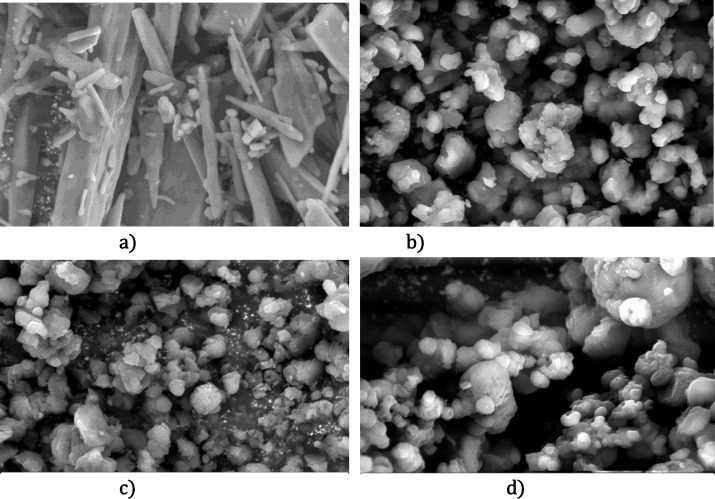
Scanning electron micrograph of a) untreated acetaminophen crystals, b) spray dried acetaminophen in absence of polyninylpyrrolidone, c) co-spray dried acetaminophen in presence of 2.5% polyninylpyrrolidone, d) co-spray dried acetaminophen in presence of 5% polyninylpyrrolidone

Scanning electron micrographs of different samples are shown in Figures 1a-d. 

**Table 2:** The onset of melting point, the enthalpy of fusion and percent of crystallinity for untreated acetaminophen and spray dried samples

Sample	Onset of Melting point	ΔH (J/g)	% Crystallinity
Untreated acetaminophen	170.6±0.2	198.9±3.3	100
Spray dried acetaminophen in absence of PVP	169.2±0.3	169.9±4.1	85.42
Co-spray dried acetaminophen + 1.25% PVP	167.4±0.1	116.6±2.7	58.60
Co-spray dried acetaminophen + 2.5% PVP	165.1±0.4	94.6±3.2	47.55
Co-spray dried acetaminophen + 5% PVP	164.4±0.3	72.2±5.2	36.31

***DSC studies***

**Figure 2.**
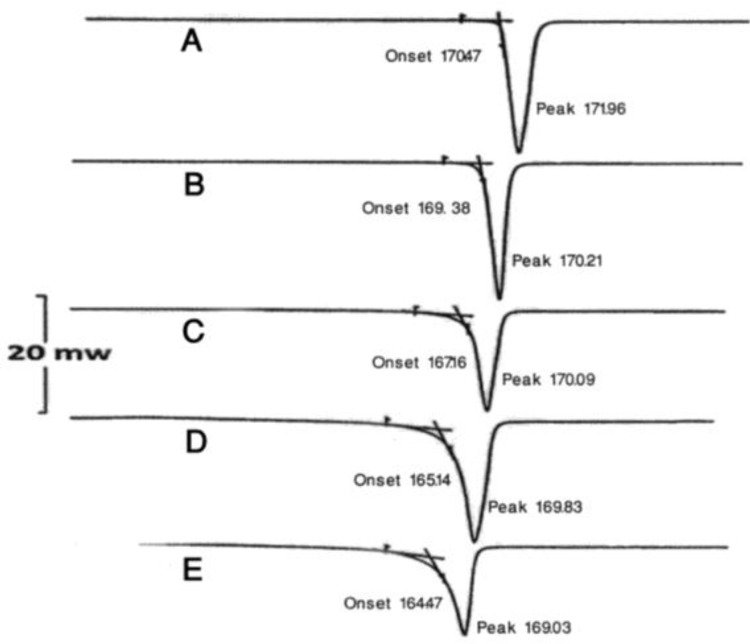
DSC thermograms of: A) untreated acetaminophen and spray dried samples obtained in presence of: B) 0%, C) 1.25%, D) 2.5% and E) 5% polyninylpyrrolidone

Figure 2 shows DSC thermograms of untreated acetaminophen and spray dried samples. In Table 2 the values ​​of enthalpy of fusion and relative amounts of crystallinity calculated based on equation 2 are presented. 

***XRPD studies***

**Figure 3:**
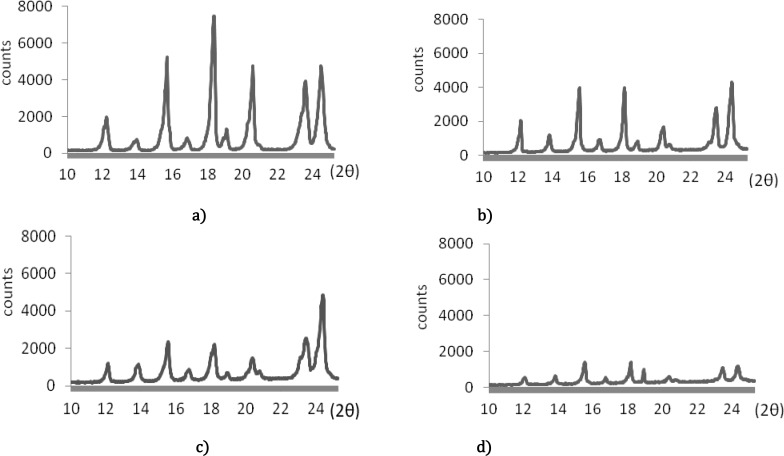
XRPD spectra of a) untreated acetaminophen and spray dried samples obtained in the presence of b) %1.25 polyninylpyrrolidone, c) %2.5 polyninylpyrrolidone, and d) 5% polyninylpyrrolidone

The XRPD spectra of untreated acetaminophen and spray dried samples are shown in Figures 3 a-d. The area under a peak exactly at 18° 2θ and the relative amount of crystallinity based on equation 3 is presented in Table 3.

***Dissolution test***

The MDT values for untreated acetaminophen and spray dried samples are presented in Table 4. 

***Compaction study***

**Figure 4:**
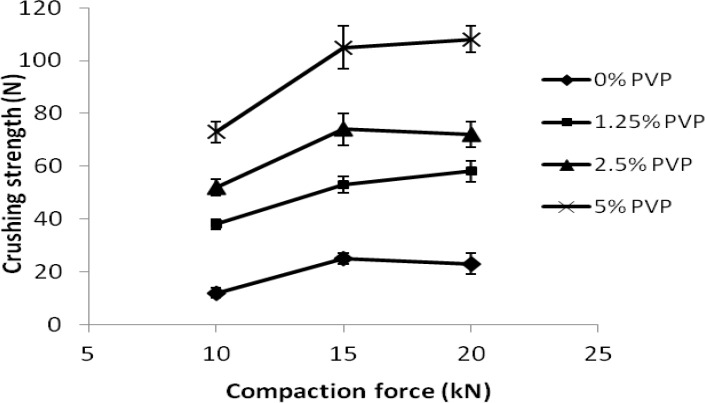
Effect of compression force on crushing strengths of tablets made from spray dried acetaminophen samples obtained in presence of different amounts of PVP

The influence of compression force on crushing strengths of tablets made from spray dried acetaminophen obtained in the absence or presence 

of PVP are shown in Figure 4. Effect of compression force on the crushing strengths of tablets made from physical mixtures of acetaminophen with different contents of PVP (2.5% and 5% w/w) are also presented in Table 5.

## Discussion

The percentage yield of spray drying process varied between 35.7% and 65.1%, depending on the concentration of PVP (Table 1). The lowest yield was obtained for the samples spray dried from solution containing no PVP and the highest yield was obtained for the samples produced from solution containing 5% PVP. Regarding Table 1, it can be deduced that there is a direct relationship between the percentage of yield and the concentration of PVP used in the solutions.

This could be explained by the data of particle size analysis. Table 1 clearly shows that the mean particle size of samples increased with the increase in PVP concentration. For instance, the mean particle size of spray dried samples increased from 11 to 18.5 µm when the concentration of PVP increased from 0 to 5% w/w. The increase in mean particle size of the spray dried samples is probably due to the increase in the solid content of liquid feed, and also increase in viscosity of the solution. In addition, the presence of PVP could induce the adhesion of particles to each other and promote agglomerate formation (22, 23). Therefore, the enhanced yield with increase in PVP concentration could be due to increase in particle size of the samples. Generally, during spray drying, increase in mean particle size of the samples minimizes the escape of suspended fine particles in air from cyclone.

**Table 3:** The relative amount of crystallinity for acetaminophen samples obtained based on the area under a curve in XPRD spectra

Sample	Area under a peak at 18°2θ	%Crystallinity
Untreated acetaminophen	2411.76	100
Co-spray dried acetaminophen + %1.25 PVP	1581.89	65.59
Co-spray dried acetaminophen + %2.5 PVP	1365.99	56.63
Co-spray dried acetaminophen + %5 PVP	790.34	32.77
		
		

Scanning electron micrographs of different samples are shown in Figures 1a-d. Untreated acetaminophen crystals exhibit acicular shape with 33µm length and 7µm width in average (Figure 1a). Spray drying of hydroalcoholic solution in the absence of PVP changed the morphology of acetaminophen particles to agglomerated structure containing grain-like microparticles with an average size of 4 µm (Figure 1b). The small sizes for these particles could be due to the nature of the hydroalcoholic vehicle used. Ethanol could reduce the surface tension of the solution and may contribute to formation of small size spray droplets. This could lead to formation of spray dried particles with the size of 3-4 µm. Samples obtained from the solutions containing different amounts of PVP also produced agglomerated structure containing spherical microparticles with an average size of 3 µm (Figures 1 c and d). Such changes in particle morphology have been previously reported for spray dried samples of acetaminophen. However, in those studies high amounts (more than 50%) of polymers such as sodium carboxymethylcellulose or chitosan were used (15, 22) whilst in the present study only small amounts of PVP (maximum 5%) were used. 

DSC and XRPD studies have been widely used to determine the solid states characteristics and crystallinity of different drugs in combination with different carriers (24, 25).

Figure 2 shows DSC thermograms of untreated acetaminophen and spray dried samples. In Table 2 the values ​​of the enthalpy of fusion and relative amounts of crystallinity calculated based on equation 2, are presented. Thermal analysis technique has been widely used to study the solid state characteristics of spray dried particles (15, 26, 27). It was reported that the area enclosed by integral of melting endotherm in DSC scans can be used for quantification of crystalline and amorphous content of a drug (29). Also, DSC was used for quantitative analysis of the crystallinity of indomethacin in binary systems (28). Figure 2 clearly illustrates that all samples showed a sharp melting peak with flat baseline which indicated that no events such as hydration, solvation or polymorphic transition had occurred during spray drying process. However, Table 2 indicates that the onsets of melting points and enthalpies of fusion of spray dried acetaminophen samples decreased by  1.8-6.2°C and 29-126.7 J/g, respectively, as compared to untreated acetaminophen samples. The reduction in the enthalpy of fusion and onset of melting point may be attributed to the presence of amorphous regions in particles due to weakening and disruption of crystal lattice and order. Garekani *et al* reported that crystallization of acetaminophen from a mixture of water and ethanol in the presence of PVP resulted in a reduction in melting point and enthalpy of fusion of samples (16). Table 2 clearly shows a dramatic reduction in crystallinity of acetaminophen following the spray drying process in the presence of PVP. Samples obtained in the presence of 5% w/w PVP exhibited 63.7% reduction in crystallinity. 

The XRPD spectra of untreated acetaminophen and spray dried samples are shown in Figures 3 a-d. These figures exhibit essentially similar diffraction patterns (2θ values) for all samples suggesting that spray dried particles did not undergo any structural modifications (Figure 3). However, a major reduction in relative intensities of their peaks (particularly samples containing 2.5 and 5% PVP) may be due to reduction in crystallinity and presence of amorphous state in the samples. It has been reported that PVP is a strong crystal growth inhibitor for acetaminophen (30). It was also demonstrated that there is a potential binding between acetaminophen and PVP in their aqueous solutions via hydrogen bonding (19). Therefore, it is expected that in the presence of higher concentration of PVP, acetaminophen particles with less crystallinity are produced. The area under a peak exactly at 18°2θ and the relative amount of crystallinity based on equation 3 are presented in Table 3. These results clearly show that the spray drying caused a major reduction in crystallinity of acetaminophen. The spray dried samples obtained in the absence of PVP exhibited about 35% reduction in crystallinity. Increase in the amount of PVP in the samples decreased the crystallinity to more extent so that the co-spray dried particles obtained in the presence of 5% PVP, exhibited only 32% crystallinity. In a similar study, XRPD technique was used to quantify the relative crystallinity of acetaminophen in co-precipitated or co-crystallized with PVP obtainedfrom different solvents (21). It was concluded that along with the increase in the amount of PVP, the degree of crystallinity dramatically decreased. The degree of crystallinity of acetaminophen in the solid dispersions was 1.99-76.16%, based on the PVP content. 

Overall, the results of DSC and XRPD studies were in good agreement and both studies showed that partially amorphous or semi-crystalline acetaminophen could be obtained following a spray drying process of acetaminophen from hydroalcoholic solutions in the presence of PVP. The production of partially amorphous acetaminophen particles are explained in following paragraphs.

Rapid evaporation rate of vehicle during spray drying due to nature of hydroalcoholic solvent and the use of high inlet temperature (160°C) facilitated the rapid solidification of spray droplets and contributes to production of amorphous phase. As it was mentioned above, PVP is a strong crystal growth inhibitor for acetaminophen, therefore, it was most likely that during spray drying from hydroalcoholic solution, rapid solidification of acetaminophen droplets in the presence of PVP changed the morphology and solid state characteristics of acetaminophen crystals and reduced the crystallinity of obtained particles. However, spray drying process itself could also lead to formation of the amorphous parts in the obtained particles mainly by rearrangement of the physical state. Takahashi *et al*

reported that spray drying of acetaminophen with high ratios of chitosan produced completely amorphous state (15). It has been reported that spray drying of valdecoxib and PVP K30 (at ratio 1:1 or more) from methanol produced amorphous solid (27). Production of amorphous lactose using spray drying technique in the presence of polyethylene glycol has been reported by Corrigan *et al* who used DSC and XRPD to show the presence of amorphous state in spray dried samples (2). 

The preliminary stability studies for particles stored 6 months at ambient condition showed no significant changes in their DCS and XPRD spectra, indicating no significant changes in their crystallinity (data are not shown). However, the stability studies at various temperatures and relative humidity are being evaluated and the results will be published later.

The MDT values for untreated acetaminophen and spray dried samples are presented in Table 4. This table clearly indicates that there is a marked enhancement in the dissolution rate of spray dried samples specially for those obtained in the presence of PVP, compared to untreated acetaminophen. Untreated acetaminophen showed a MDT of 19.7 min while the particles obtained in the presence of 5% PVP exhibited a MDT of 2.8 min. The enhancement in dissolution rate of spray dried particles may be explained by three different reasons:

1- Reduction in crystallinity of acetaminophen and formation of amorphous phase in spray dried samples (Tables 2 and 3) tend to increase the dissolution rate of acetaminophen. Amorphous form is a high-energy state that would improve the dissolution rate of low soluble drug substances (27). 

**Table 4:** The calculated MDT± SD for different acetaminophen samples

sample	Untreated acetaminophen	Spray dried acetaminophen	Co-spray dried acetaminophen + 1.25% PVP	Co-spray dried acetaminophen + 2.5% PVP	Co-spray dried acetaminophen + 5% PVP
MDT (min)	19.7±2.3	10.9±1.7	6.8±1.1	5.2±1.4	2.8±0.6

**Table 5:** Effect of compression force on the crushing strengths of tablets made from physical mixtures of acetaminophen and different amounts of PVP (2.5% and 5% w/w):

Compression force (kN)	Crushing strength (N)	
	2.5% w/w PVP	5% w/w PVP
5	*	3±1
10	*	6±2
15	*	5±2

very weak tablets with no measurable hardness

Several studies have already indicated that the presence of amorphous state in particles enhances the dissolution rate of some drugs (14, 31).

2- Reduction in particle size. Scanning electron micrographs (Figure 1a- d) shows that untreated acetaminophen particles have acicular shape with 33 µm length and 7 µm width. The particles obtained in the absence or presence of 2.5% or 5% w/w PVP had an agglomerated structure consisting of microparticles with diameter of 3-4 µm. Presence of these microparticles in the samples could be another reason for the dissolution enhancement of acetaminophen from spray dried samples. A decrease in particle size increases the surface area of acetaminophen particles exposed to the dissolution media and subsequently increases the dissolution rate.

3- The presence of PVP in the samples increased the wetability. PVP is a water soluble polymer and increases the wetability of acetaminophen particles. Table 4 clearly shows that with increase in the amount of PVP in particles, the dissolution rate increased. Similar results reported by Garekani *et al* (18) showed that crystallized acetaminophen in the presence of small amount of PVP exhibited a marked enhancement in dissolution rate of acetaminophen and this was attributed to the adsorption of PVP on the surface of acetaminophen crystals. 

The influence of compression force on crushing strengths of tablets made from spray dried acetaminophen obtained in the absence or presence of PVP are shown in Figure 4. Effect of compression force on the crushing strengths of tablets made from physical mixtures of acetaminophen with different contents of PVP (2.5% and 5% w/w) are also presented in Table 5.

Compression of untreated acetaminophen at all compression forces produced extremely weak tablets with no measurable crushing strengths and with a high tendency to cap. Figure 4 shows that the spray dried acetaminophen particles especially those obtained in the presence of PVP exhibited an obvious improvement in their compaction properties compared to untreated acetaminophen. Acetaminophen particles which were spray dried in the absence of PVP exhibited a minor enhancement in their compaction properties and produced tablets with maximum hardness of 25 N with tendency to cap. However, co-spray dried samples obtained in the presence of PVP exhibited a major improvement in their compaction properties and produced tablets with excellent crushing strength and lack of tendency to cap. Figure 4 clearly shows that an increase in the amount of PVP in spray dried samples resulted in a profound increase in the crushing strength of tablets. The high crushing strength of tablets is indicative of stronger interparticulate bonding between particles. This may be attributed to the presence of PVP in particles which is known as a good binder, or may be due to the solid state characteristics of these particles. Data related to crushing strengths of physical mixtures of sieved fractions of PVP and acetaminophen compressed at different compression forces (Table 5), clearly indicate that the crushing strengths of tablets even at 5% w/w PVP content were less than 10 N, whereas particles spray dried in the presence of 5% PVP produced tablets with crushing strengths more than 100 N (Figure 4). Therefore, these results indicate that improvement in compaction properties of spray dried acetaminophen obtained in the presence of PVP was due to the solid state characteristics and the presence of amorphous state in these particles and was not only due to the presence of PVP. However, both the agglomerate structure and porosities of the particles might have contribution to the improved compaction properties. The presence of amorphous state in pharmaceutical powder plays an important role in their compactibility. Berggren *et al* (4) and Corrigan *et al* (2) showed that spray drying of lactose and PVP or polyethylene glycol produced amorphous composite particles with improved compaction properties. Improvement in compaction properties of acetaminophen spray dried in the presence of some carbohydrates such as maltodextrine has been attributed to the presence of maltodextrine and agglomerate structure of particles and was not due to any polymorphic transition (32).

## Conclusion

The results of this study showed that spray drying of acetaminophen from hydroalcoholic solutions (25% v/v ethanol/water) in the presence of small amounts of PVP (maximum 5% w/w based on acetaminophen weight) produced partially amorphous particles with improved dissolution and excellent compaction properties. Acetaminophen particles obtained in the presence of PVP had agglomerated structure consisting of spherical microparticles with the size of 3-4 µm. 

DSC and XRPD experiments indicated a marked reduction in crystallinity of spray dried particles especially for those containing 5% w/w PVP. 
